# Grazer-induced morphological defense in *Scenedesmus obliquus* is affected by competition against *Microcystis aeruginosa*

**DOI:** 10.1038/srep12743

**Published:** 2015-07-30

**Authors:** Xuexia Zhu, Jun Wang, Yichun Lu, Qinwen Chen, Zhou Yang

**Affiliations:** 1 Jiangsu Key Laboratory for Biodiversity and Biotechnology, School of Biological Sciences, Nanjing Normal University, 1 Wenyuan Road, Nanjing 210023, China

## Abstract

The green alga *Scenedesmus* is known for its phenotypic plasticity in response to grazing risk. However, the benefits of colony formation induced by infochemicals from zooplankton should come with costs. That is, a tradeoff in benefit-to-cost ratios is likely under complex environmental conditions. In this study, we hypothesized that the coexistence of *Scenedesmus* and its competitors decreases the formation of anti-grazer colonies in *Scenedesmus*. Results demonstrated that the presence of a competitor *Microcystis aeruginosa* inhibited inducible defensive colony formation of *Scenedesmus obliquus*, and the established defensive colonies negatively affected the competitive ability of *S. obliquus*. The proportion of induced defensive colonies in cultures was dependent on the relative abundance of competitors. Under low competition intensity, large amount of eight-celled colonies were formed but at the cost of decreased competitive inhibition on *M. aeruginosa*. By contrast, defensive colony formation of *S. obliquus* slacked in the presence of high competition intensity to maintain a high displacement rate (competitive ability). In conclusion, *S. obliquus* exhibited different responses to potential grazing pressure under different intensities of competition, i.e., *Scenedesmus* morphological response to grazing infochemicals was affected by competition against *Microcystis*.

The production of different phenotypes from a single genotype in response to environmental conditions is termed “phenotypic plasticity”, which is common in organisms^1^,^2^,^3^. Phenotypic plasticity is an adaptation to natural environmental selection pressures^4^. The ability to adapt to multiple environments is very important for the evolutionary success of organisms^4^,^5^. Among the biotic and abiotic factors that alter the expression of organisms’ genotypes, predation pressure plays an important role. Organisms may respond to the selective pressure of predation by producing defensive phenotypes against predators. For example, to avoid being attacked by lake crabs, blue mussels *Mytilus edulis* are induced by predation cues to increase shell thickness, enhance abductor muscle strength, and produce more byssal threads^6^. Similarly, *Daphnia pulex* produces neck teeth on the dorsal anterior margin of the head in the presence of the predator *Chaoborus americanus*^7^. Predation-induced phenotypic plasticity is also prevalent in plants. Some plants produce toxins and defensive proteins or emit volatiles to discourage herbivores^8^.

Competition and predation are considered the major selective pressures that shape the composition of pelagic photoautotrophic communities^9^. In general, edible phytoplankton have less competitive advantage than resistant phytoplankton under high grazing pressure^10^. Many phytoplankton species have developed morphological and chemical defenses or other mechanisms to resist the attack of predators and avoid loss of biomass caused by grazing^11^,^12^. As for unicellular algae, which are smaller than their predatory enemies, one effective way to resist grazing threat is to increase in size^13^. Induced colony formation in the green alga *Scenedesmus* spp. is a typical case. Upon detection of infochemicals released by herbivores such as *Daphnia, Scenedesmus* populations become dominated by four- and eight-celled colonies instead of unicells^14^. *Scenedesmus* populations that are dominated by colonies can effectively deter feeding by small grazers such as *Daphnia cucullata*; therefore, induced colony formation is an efficient strategy for *Scenedesmus* to escape grazing by predators with a diet limited by the prey size^15^.

However, large *Scenedesmus* colonies experience a higher risk of sedimentation than unicells^16^. The light dependence of pelagic photoautotrophic organisms limits their size^17^. Large algal particles without additional buoyancy mechanisms, such as gas vesicles, may have limited capacity to suspend in the euphotic zone. Increase in colony size also decreases their surface-to-volume ratios. Algae absorb nutrients through the cellular membrane, and large surface-to-volume ratios benefit nutrition uptake and light capture^17^,^18^. Under turbulent diffusion, phytoplankton species are mixed and coexist in water. All phytoplankton species and their competitors require almost the same nutrients from the same resource pool^19^. As a result, competition for light and nutrients will be an essential requirement for the population of phytoplankton to sustain domination in phytoplankton communities^20^,^21^. Hence, trade-offs potentially exist between the formation of large sinkable colonies (favoring defense) and maintenance of vulnerable unicells (favoring competition) in *Scenedesmus*.

The disadvantages of forming colonies, including accelerated sinking rate and decreased nutrition and light absorption, may affect the competitive ability of *Scenedesmus*. It has been recognized that organisms face trade-offs to allocate the limited resources to grow or to counteract stress^22^,^23^. The trade-off between competitive ability and resistance to predators or parasitoids is common in many species. For example, defended algae have less competitive ability than undefended species^24^,^25^.

Considering the trade-off mechanism and the high morphological plasticity of *Scenedesmus* in response to infochemicals released by grazers, we hypothesized that defense and competition operate at the expense of each other and *Scenedesmus* may make trade-offs under pressure from both predators and competitors. Accordingly, this study aimed to examine the costs and the trade-off of forming grazer-induced defensive colonies in *Scenedesmus* under different levels of inter-species competition. *Microcystis aeruginosa*, one of the most common bloom-forming cyanobacteria, was used as the competitor of *S. obliquus*. Both algae are found in freshwaters world-wide and often coexist in the same area^26^,^27^. *M. aeruginosa* has been reported to have significant allelopathic influence on co-existing phytoplankton^28^,^29^,^30^,^31^ and to produce secondary metabolites that are toxic to zooplankton^32^. In natural waters or under the grazing induction of predators^33^, *M. aeruginosa* can also form huge thousand-celled colonies, but the *M. aeruginosa* strain selected in this study was unicellular with no colony formation when exposed to *Daphnia* grazing infochemicals^34^. We used filtered test water containing infochemicals from *Daphnia magna* to simulate *S. obliquus* colony formation in the presence of competitors.

## Results

**Figure 1:**
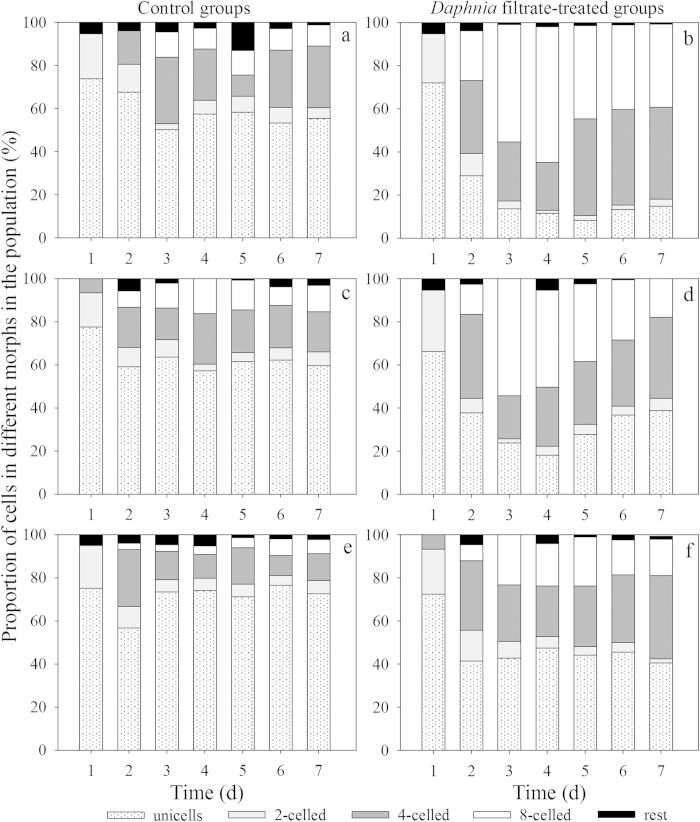
Proportions of unicells and 2-, 4-, and 8-celled ceonobia in *Scenedesmus* populations of monocultures in the absence (**a**) and presence (**b**) of *Daphnia* filtrate, in co-cultures with different initial compositions (*Scenedesmus*:*Microcystis* = 1:1, absence (**c**) or presence (**d**) of *Daphnia* filtrate; and *Scenedesmus*: *Microcystis* = 1:5 absence (**e**) or presence (**f**) of *Daphnia* filtrate during the 7-day experiment. The “rest” group represents 3-, 5-, 6-, 7-celled colonies.

**Figure 2:**
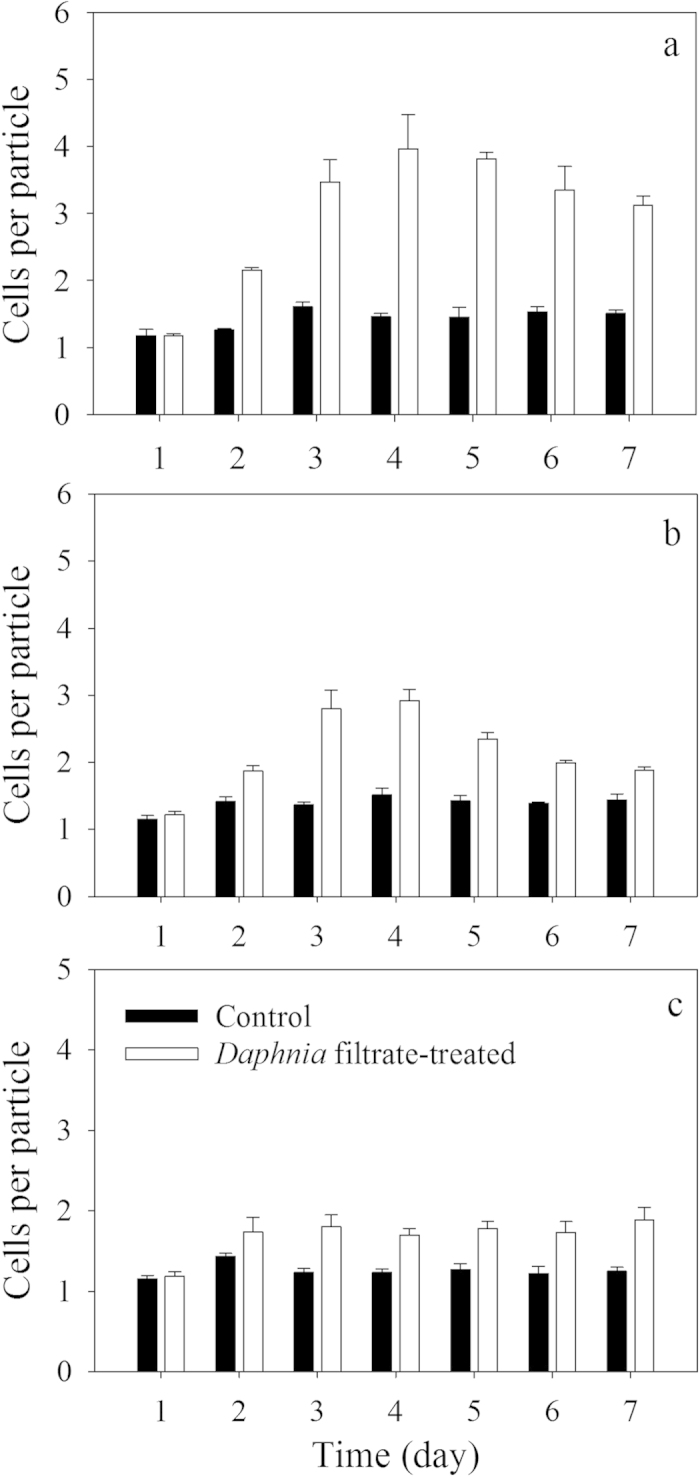
Changes in the mean number of cells per particle of *Scenedesmus* in pure cultures of *Scenedesmus* (panel a) and in co-cultures of *Scenedesmus* and *Microcystis* with different initial compositions (panel b: *Scenedesmus*: *Microcystis* = 1:1; panel c: *Scenedesmus* : *Microcystis* = 1:5) in the absence (black bars) and presence (white bars) of *Daphnia* filtrate. Vertical lines represent 1SE.

Monocultures of *S. obliquus* in the control groups were dominated by unicells over the 7 d period, with less than 12% large eight-celled coenobia (Fig. 1a). The morphology of *S. obliquus* monocultures changed drastically under exposure to *Daphnia* filtrate compared with the control. The proportion of unicells decreased rapidly from day 2, and four- and eight-celled coenobia formed (Fig. 1b). Colony formation reached its maximum on day 4 (Fig. 2a), and the proportion of four- and eight-celled coenobia increased to approximately 85%. On day 5, the proportion of eight-celled coenobia decreased from more than 60% to approximately 40%. The mean number of cells per particle in the control groups remained below two during the 7 d period. In this experiment, the *S. obliquus* population of each culture was composed by unicells and two-, four-, and eight-celled colonies; a few three-, five-, six-, and seven-celled colonies were also present, but coenobia larger than eight cells were not observed.

In co-cultures of the control groups, the *S. obliquus* populations were dominated by unicells as the monocultures (Fig. 1c,e). However, the defensive morphological features of *S. obliquus* induced by *Daphnia* filtrate were significantly altered when *M. aeruginosa* was added as a competitor to the *S. obliquus* cultures. The induced colony formation of *S. obliquus* decreased in the presence of *M. aeruginosa* (Fig. 2b,c). The mean number of cells per particle of *S. obliquus* in the *Daphnia* filtrate-treated groups co-cultured with 1-fold *M. aeruginosa* reached its maximum on day 4 (Fig. 2b), which was similar to the pattern of the *Daphnia* filtrate-treated monocultures (Fig. 2a). By contrast, the colony size decreased rapidly from day 5 and unicells became dominant again. The proportion of unicells was approximately 40% on day 7 (Fig. 1d), which was significantly higher than that in the monocultures (Fig. 1b). However, in the co-cultures with 5-fold *M. aeruginosa*, the average colony size of *S. obliquus* was smaller than two cells even though colonies of *S. obliquus* were significantly induced by *Daphnia* filtrate (compared with the control group in this experiment, *F *= 67.398, *p* < 0.001) (Fig. 2c). Unicells accounted for approximately 40–48% of the total populations from day 2 to the end of the experiment (Fig. 1f). The competitive effect of *M. aeruginosa* emerged on day 3. Formation of four- and eight-celled large coenobia of *S. obliquus* ceased (Fig. 1d,f). In the control groups, *M. aeruginosa* was unicellular or paired cellular. *M. aeruginosa* exposed to *Daphnia* filtrate failed to form colonies and were found predominantly as unicells and paired cells.

**Figure 3:**
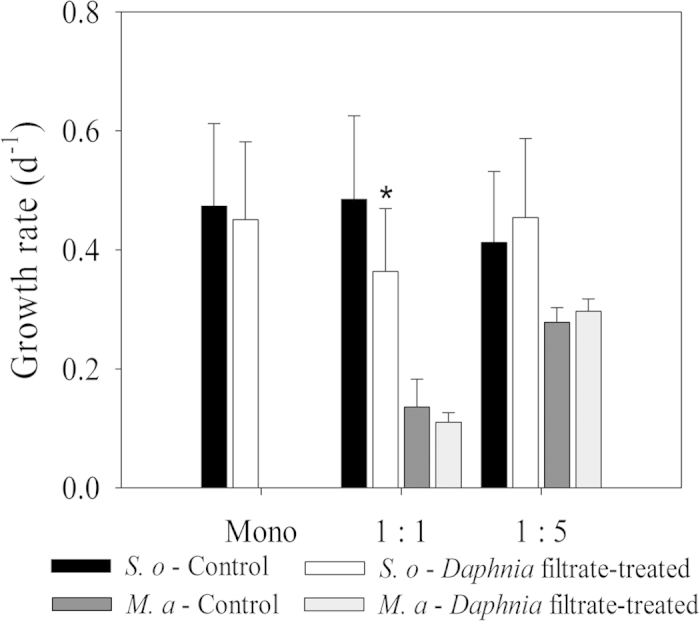
Growth rates of *S. obliquus* and *M. aeruginosa* populations incubated under different conditions during the 7-day experiment. Vertical lines represent 1 SE, short lines with asterisk above two bars represent significant difference (*p* < 0.05) within groups.

Growth of *S. obliquus* decreased significantly in the *Daphnia* filtrate-treated co-cultures with 1-fold *M. aeruginosa* when compared to the control monoculture group (Fig. 3; one-way ANOVA; *p *= 0.001). Growth rates of *S. obliquus* in other four group cultures showed no significant differences compared with the control monoculture group. Two-way ANOVA was conducted to analyze the influence of *Daphnia* filtrate and the density of competitors on the growth rates of *S. obliquus* and *M. aeruginosa* (Table 1). Results showed that *Daphnia* filtrate had a strong effect on the growth rates of *S. obliquus* and significant interaction was detected between these two factors. *M. aeruginosa* had significantly higher growth rates in co-cultures with higher initial cell density. *Daphnia* filtrate had no significant effect on *M. aeruginosa* growth.

**Figure 4:**
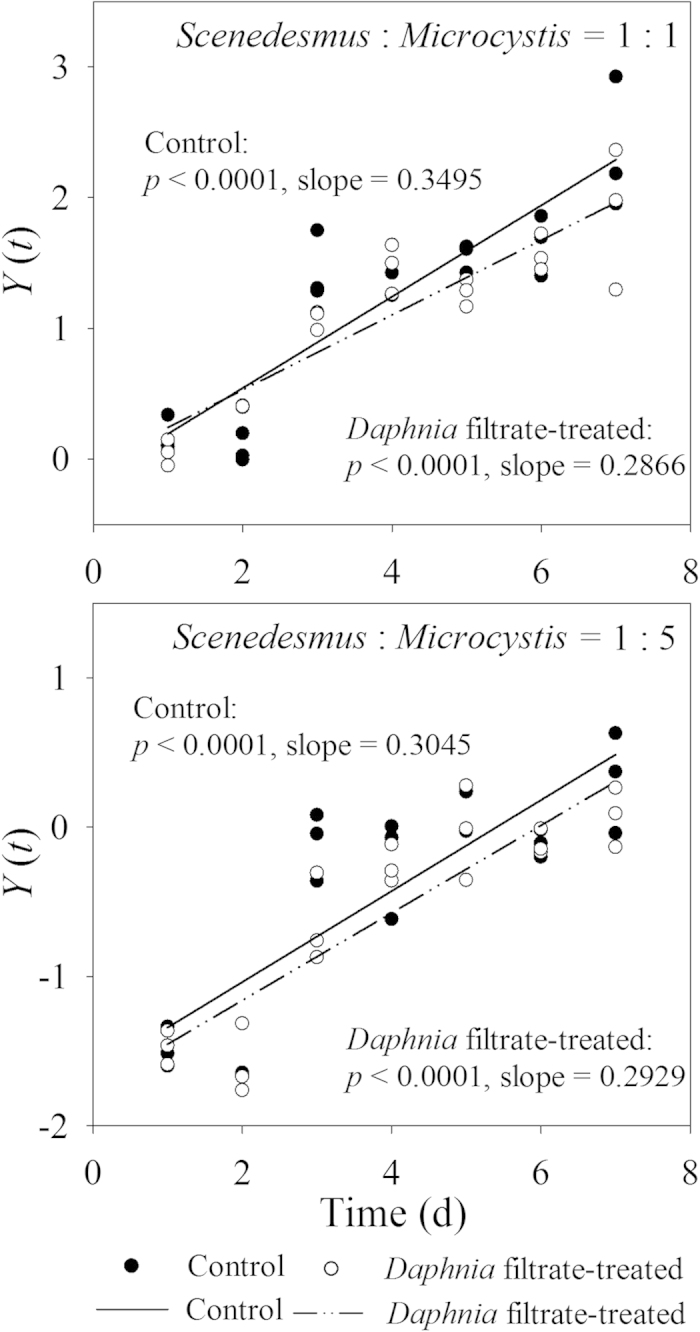
Comparison of displacement rates for *Scenedesmus* between controls and *Daphnia* filtrate-treated groups in the presence of *Microcystis* with different initial densities. Linear regressions were separately fitted for different treatments.

Separate linear regressions were fitted for all co-cultures to compare the displacement of *M. aeruginosa* by *S. obliquus* between the control and *Daphnia* filtrate-treated groups (Fig. 4). The slopes of *Y*(*t*) versus *t* were positive in all cultures, indicating that *S. obliquus* could displace *M. aeruginosa* in both experiments. In co-cultures with the same initial cell densities of both algae, the slope decreased in the *Daphnia* filtrate-treated group compared with the control, but the decrease was not statistically significant (*F *= 1.723, *p *= 0.196). The displacement rates during days 3 to 7 were further analyzed, because colony formation induced by *Daphnia* infochemicals peaked on day 3. Since multicellular colonies have lower surface area-to-volume ratios than unicells, nutrition uptake of *S. obliquus* may be affected under competition by *M. aeruginosa* and the potential costs may emerge after day 3. Significant decrease was detected in the *Daphnia* filtrate-treated group (*F *= 4.789, *p *= 0.037). For co-cultures with 5-fold *M. aeruginosa*, the slopes of the control and *Daphnia* filtrate-treated groups were similar (*F *= 0.843, *p *= 0.212). Coexistence with *M. aeruginosa* decreased the formation of inducible colonies by *S. obliquus* for potential reallocation of resources in response to competition.

## Discussion

*Scenedesmus* is considered to benefit from the ability of forming plastic morphs in response to biotic and abiotic environmental factors, particularly herbivory^15^. The algal defense theory for freshwater systems predicts that edible species of algae with no or weak defensive mechanisms are more vulnerable under the selective stress of predation^35^,^36^. Resistant algae with effective defensive mechanisms can escape from being consumed and replace edible algae in algal communities^12^. However, some grazer-resistant species have slower growth rates than undefended ones^25^,^37^, so it has been suggested that resistance to herbivory could be costly. For *Scenedesmus*, the benefits of being plastic have been well documented^15^,^38^, but the direct costs and limits involved in inducible resistance still lack enough evidence. The higher sinking rates of coenobia compared with unicells might be the only indirect cost detected in multicellular *Scenedesmus*^16^. Yokota and Sterner^24^ showed that coenobial *Desmodesums subspicatus* preinduced and prolonged by *Daphnia* infochemicals had suppressed competitive ability compared to unicellular ones. Thus, it is possible that colonial morph interferes with the rapid growth of *S. obliquus* for the reduction of resource uptake owing to its decreased surface area-to-volume ratio. Therefore, we hypothesized that *Scenedesmus* formed different morphs as a trade-off between defense and competition.

In the present study, we observed a trade-off in *S. obliquus* between defense and growth in the presence of competitors. Competitive displacement rates indicated that colony formation exerted adverse effects on the growth and competitive ability of *S. obliquus* (Fig. 4). Formation of large defensive colonies is a type of adaptive phenotypic plasticity under high predation stress, but this morphology will be a liability to rapid growth of the *S. obliquus* population when the threat is relieved, and thus competition stress is increasing (Fig. 4). The *Scenedesmus* population of large defensive coenobia is eventually redominated by unicells when infochemicals in the water are eliminated^39^, and enhanced competition accelarated the deformation of colonies. This strategy presumably benefits organisms by facilitating resource allocation between defense and growth^40^. In this study, *S. obliquus* showed highly morphological plasticity in the presence of competitors with different initial densities. Under different degrees of competitive stresses, the population of *S. obliquus* balanced the formation of defensive colonies and growth of the population. *M. aeruginosa* is a common bloom-forming phytoplankton that can coexist with *Scenedesmus* in temperate eutrophic lakes^41^. In the presence of relatively low competitive pressure from *M. aeruginosa, S. obliquus* responded to the grazer infochemicals by forming a high percentage of colonies at the expense of decreased competitive exclusion rate. The significant decline in the displacement rate from day 3 observed in this study may be the remarkable evidence of cost associated with colony formation. With proliferation of both algae, inter-specific competition for resources increased. The negative effect on the competitive ability of defensive colonies might inhibit further formation of colonies, as evidenced by the gradual decrease in colony size from day 4. When the initial density of *M. aeruginosa* was increased to 5-fold, competitive stress increased, limitations of phenotypic plasticity became prominent, and weak morphological modulations were detected in *S. obliquus*. Defense to herbivory was reduced to maintain competitive superiority under high competitive stress.

As the two major selective pressures affecting phytoplankton, inter-specific competition and predation can shape the composition of algal communities^42^. Intra-specific variability provided by phenotypic plasticity is known to be the mechanism that enhances the competitive and adaptive abilities of phytoplankton under varying environmental conditions^43^,^44^. Defensive responses to predators may alter the competition between prey species^45^. In the present study, as we hypothesized, inter-specific competition was found to influence the plastic response of *Scenedesmus* to herbivory. Inducible defense is more energy-saving than constitutive defense in the long term, but both defensive mechanisms are expensive^46^. One of the main benefits of the “just-in-time” inducible defense system is effectiveness^47^. The investment in a plastic defense mechanism is more effective for organisms especially when facing multiple environmental pressures. Continuous reproduction of large colonies would affect the competitive ability of *Scenedesmus*^24^, and unicellular or small colonial forms would be favored under intense competitive environments. The failure to form typical anti-predator colonies might be due to the strong competition pressure and the sense of relative reduction in the risk of being grazed on. Because higher density of co-existing species will relieve the grazing pressure on a single species, there is no need for *S. obliquus* to form colonies that have higher sedimentation rate and lower resource acquisition at the expense of competitive ability.

Besides the possible trade-off of *S. obliquus* between forming defensive colonies and competition, there are other possible reasons for decreased colony formation in *S. obliquus* when competitors co-exist. *M. aeruginosa* may interfere with the regular response of *S. obliquus* to grazing cues, as *M. aeruginosa* can produce secondary metabolites that have inhibitory effects on photoautrophic algae^29^,^31^; these allelochemicals may interfere with the response of *S. obliquus* to *Daphnia* infochemicals. In addition, some infochemicals released by *Daphnia* may be absorbed by *M. aeruginosa* cells (despite *M. aeruginosa* not showing change in morphology), which lowers the amount of infochemicals detected by *S. obliquus* cells and thus lowers their morphological response.

In summary, the presence of a competitor *M. aeruginosa* inhibited inducible defensive colony formation of *S. obliquus*. The proportion of induced defensive colonies in cultures was dependent on the relative abundance of competitors. Under low competition intensity, large amount of eight-celled colonies were formed but at the cost of decreased competitive inhibition on *M. aeruginosa*. By contrast, defensive colony formation of *S. obliquus* slacked in the presence of high competition intensity to maintain a high competitive ability. *S. obliquus* exhibited different responses to potential grazing pressure under different intensities of competition. These results demonstrate that phenotypic plasticity allows organisms to undergo controllable phenotypic changes to reduce fitness costs by optimizing the benefit-to-cost ratios when responding to different environmental challenges.

## Methods

### Microorganisms and culture conditions

*S. obliquus* (Chlorophyta) FACHB-416 and *M. aeruginosa* (Cyanobacteria) FACHB-927 were obtained from the Freshwater Algae Culture Collection at the Institute of Hydrobiology China (FACHB-collection) and maintained in our laboratory in a modified BG-11 medium^48^ with ammonium NH_4_Cl as nitrogen source. Before the experiment, stock algae were regularly transferred to fresh media at a ratio of 0.3 d^−1^ for 10 d to maintain the logarithmic growth phase. The grazer *Daphnia magna* used to produce infochemicals in the experiment was a laboratory clone. The only food source of *D. magna* during the cultivation was *S. obliquus* grown in continuous cultures. The stock cultures of *S. obliquus, M. aeruginosa*, and *D. magna* were all stored in a climate-controlled chamber at 25 °C under fluorescent light at 40 μmol quanta·m^−2^·s^−1^ in a 14:10 h light-dark cycle. All experiments were conducted under these conditions.

### Preparation of control and *Daphnia* filtrates

Since the infochemicals that evoke anti-grazing colonies of *Scenedesmus* were demonstrated to be associated with the grazing progress and linked to alga-grazer interaction^11^,^49^, it is necessary to use “*Daphnia* feeding *Scenedesmus*” to obtaining the infochemicals. Test water containing infochemicals from *D. magna* was obtained as previously described^15^,^50^. In brief, *D. magna* was washed to remove surface contamination and allowed to evacuate their guts in sterile distilled water for at least 12 h and then incubated at a density of 300 ind·L^−1^ with sufficient *S. obliquus* as food. After 24 h, *D. magna* was removed, and the cultures were filtered through a 0.10 μm membrane filter (Millipore Corporation, USA). The filtrates served as the treatment media containing infochemicals released by *Daphnia*. Cultures of *Scenedesmus* without *Daphnia* were filtered as well and used as the control media. NH_4_Cl was used as nitrogen source to easily adjusting the nitrogen levels of filtrates since the nitrogenous metabolites of the crustacean is ammonia. Appropriate ammonium and phosphate were added to the control filtrate according to the measured concentrations of nitrogen and phosphorus in the *Daphnia* filtrate, and thus the nitrogen and phosphorus concentrations in the treatment and control media were adjusted to the same levels.

### Induction experiment under competition with *Microcystis*

**Figure 5:**
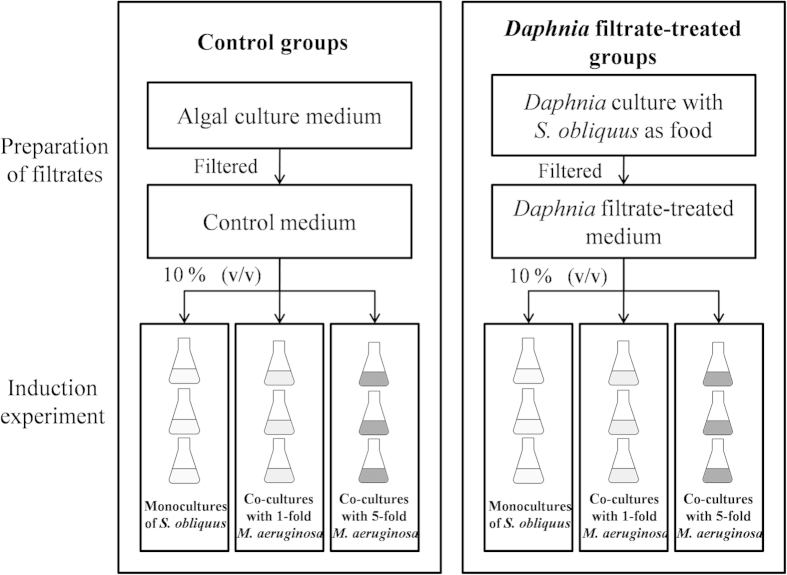
Flow diagram of the experimental design .

A flow diagram of experimental design was shown in Fig. 5. Considering the differences in cell size between *S. obliquus* and *M. aeruginosa*, we executed the experiment using two initial density proportions (*Scenedesmus*: *Microcystis* = 1:1 and 1:5) with 10% filtered *S. obliquus* culture medium or *Daphnia* filtrate. The initial algal density of *S. obliquus* in each culture was approximately 1.0 × 10^5^ cells·mL^−1^, and the initial algal densities of *Microcystis* were approximately 1.0 × 10^5^ and 5.0 × 10^5^ cells·mL^−1^, respectively. In the experiments, algal suspensions were transferred to 250 mL cellulose-plug stoppered Erlenmeyer flasks containing 150 mL of medium. Six treatments were carried out, and each treatment was run in triplicate. To avoid sedimentation, the algal cultures were manually shaken twice a day.

### Microscopic observation

Samples (1 mL) were collected daily and preserved in Lugol’s fixative (2%). Algal densities and different-celled colony distributions were counted under a microscope (Olympus 6V20WHAL; Tokyo, Japan) using a hemocytometer(Qiujing HT-042; Shanghai, China). Different algal particles (unicells, two-celled, four-celled, eight-celled, and the rest) were counted, and the mean proportions of cells in different morphs were calculated. The numbers of cells per particle were calculated by dividing the total cell number by the number of particles. Growth rates of *S. obliquus* and *M. aeruginosa* were determined as the slope of logarithm abundance versus time^51^. To reveal the potential cost of grazer-induced defenses in *Scenedesmus* under competitors, the competitive displacement rate was adapted^52^,^53^ as an index in this study. The natural log of the ratio of the abundances of the two algae, *Y*(*t*) = ln[N_*Scenedesmus*_(*t*)/N_*Microcystis*_(*t*)], was calculated and regressed against time (d). The displacement rates for *Scenedesmus* were the slopes of the linear regressions^24^.

### Statistical analysis

Data values are expressed as means of 3 replicates ± SE. Colony formation, proportions of different celled populations, and growth rates were compared by two-way ANOVA, with the presence/absence of the competitor and *Daphnia* filtrate as the two factors. Significant differences between treatments were detected by *t*-test. Statistical analyses were performed using SigmaPlot 11.0. The differences between slopes of the linear regressions of *Y*(*t*) versus *t* were analyzed by ANCOVA in SPSS 10.0.

## Additional Information

**How to cite this article**: Zhu, X. *et al*. Grazer-induced morphological defense in *Scenedesmus obliquus* is affected by competition against *Microcystis aeruginosa. Sci. Rep*. **5**, 12743; doi: 10.1038/srep12743 (2015).

## Figures and Tables

**Table 1:** Summary of two-way ANOVA of the effects of initial density of *M.aeruginosa* and *Daphnia* filtrate on the growth rate of *S. obliquus* and *M. aeruginosa*.

Algae	Source of Variation	DF	SS	MS	F	P
*S. obliquus*	Initial density of *M. aeruginosa*	2	0.00466	0.00233	2.281	0.145
	*Daphnia* filtrate	1	0.00518	0.00518	5.071	0.044
	Initial density of *M. aeruginosa *× *Daphnia* filtrate	2	0.0203	0.0102	9.961	0.003
*M. aeruginosa*	Initial density of *M. aeruginosa*	1	0.0811	0.0811	30.894	<0.001
	*Daphnia* filtrate	1	0.0000317	0.0000317	0.0121	0.915
	Initial density of *M. aeruginosa *× *Daphnia* filtrate	1	0.00145	0.00145	0.554	0.478
